# Supplementary material for: High Trunk Truncation as a Potential Sustainable Management Option for Asian Longhorned Beetle on Salix babylonica
Source: Insects. 2024 Apr 16;15(4):278. doi: 10.3390/insects15040278 (PMC11050171; doi:10.3390/insects15040278)
Supplement: Supplementary file 1 [file insects-15-00278-s001.zip › insects-2930416-supplementary.pdf]

Supplementary Materials:

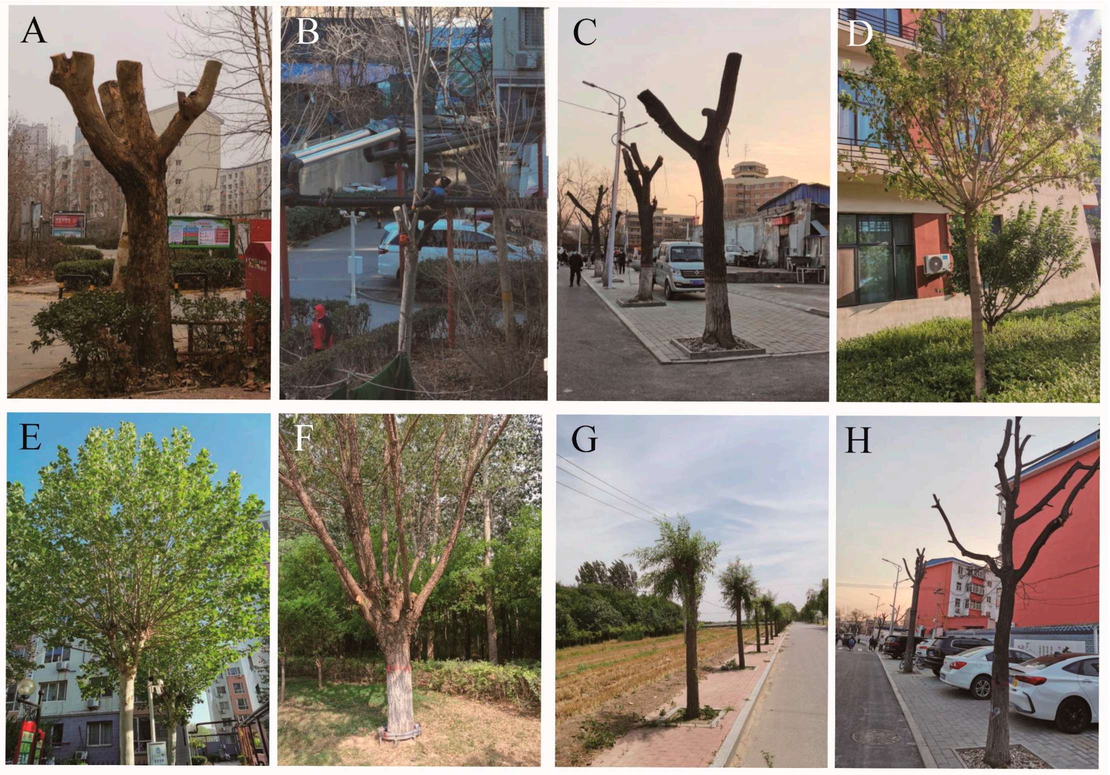

Figure S1. Different tree species managed by high trunk truncation in China. A: Pruned crown of *Platanus orientalis*. B: Pruned crown of *Poplar*. C: Pruned crown of Willow. D: *A. negundo* 3 years after high trunk truncation. E: *Platanus orientalis* after high trunk truncation in the summer of the current year. F: *S. babylonica* 7 years after high trunk truncation. G: *S. babylonica* were managed by high trunk truncation in the Mancheng. H: Pruned crown of *Sophora japonica*.

Table S1. The bark thickness of different diameter of *S. babylonica*

Sample \ Diameter	0.5 cm	1 cm	2 cm	3 cm	4 cm
1	0.71	0.86	1.43	2.43	3.13
2	0.75	0.82	1.29	2.33	2.87
3	0.64	0.89	1.35	2.16	3.07
4	0.68	0.77	1.14	2.07	3.13
5	0.57	1.06	1.52	2.24	3.23
6	0.72	1	1.17	2.63	3.32
7	0.58	0.75	1.33	2.38	3.38
8	0.89	0.7	1.1	2.32	3.22
9	0.6	0.96	1.26	2.64	3.29
10	0.73	0.86	1.28	2.7	2.97
11	0.68	0.89	1.12	2.23	3.15
12	0.58	0.76	1.11	2.68	2.99
13	0.57	0.75	1.25	2.31	2.84
14	0.64	0.78	1.55	2.36	2.98
15	0.55	1.03	1.31	2.41	3
16	0.51	0.98	1.21	2.37	3.11
17	0.57	0.95	1.48	2.31	3.24
18	0.61	0.79	1.46	2.33	3.04
19	0.6	0.88	1.24	2.35	3.1
20	0.69	0.85	1.32	2.11	2.96
